# Orchestrated Movement Sequences and Shape-Memory-like Effects in Pine Cones

**DOI:** 10.3390/plants13152078

**Published:** 2024-07-26

**Authors:** Martin Horstmann, Thomas Speck, Simon Poppinga

**Affiliations:** 1 Department of Animal Ecology, Evolution and Biodiversity, Ruhr-University Bochum, 44780 Bochum, Germany; 2 Plant Biomechanics Group, Botanical Garden, University of Freiburg, 79104 Freiburg im Breisgau, Germany; thomas.speck@biologie.uni-freiburg.de; 3 Cluster of Excellence livMatS @ FIT—Freiburg Center for Interactive Materials and Bioinspired Technologies, University of Freiburg, 79110 Freiburg, Germany; 4 Botanical Garden, Department of Biology, Technical University of Darmstadt, 64287 Darmstadt, Germany

**Keywords:** hygroscopic movement, shape-memory-like effects, pine cone movement, bilayer actuation, functional robustness, tissue mechanics, water uptake manipulation, time lapse

## Abstract

Hygroscopic seed-scale movement is responsible for the weather-adaptive opening and closing of pine cones and for facilitating seed dispersal under favorable environmental conditions. Although this phenomenon has long been investigated, many involved processes are still not fully understood. To gain a deeper mechanical and structural understanding of the cone and its functional units, namely the individual seed scales, we have investigated their desiccation- and wetting-induced movement processes in a series of analyses and manipulative experiments. We found, for example, that the abaxial scale surface is responsible for the evaporation of water from the closed cone and subsequent cone opening. Furthermore, we tested the capability of dry and deformed scales to restore their original shape and biomechanical properties by wetting. These results shed new light on the orchestration of scale movement in cones and the involved forces and provide information about the functional robustness and resilience of cones, leading to a better understanding of the mechanisms behind hygroscopic pine cone opening, the respective ecological framework, and, possibly, to the development of smart biomimetic actuators.

## 1. Introduction

**Figure 1:**
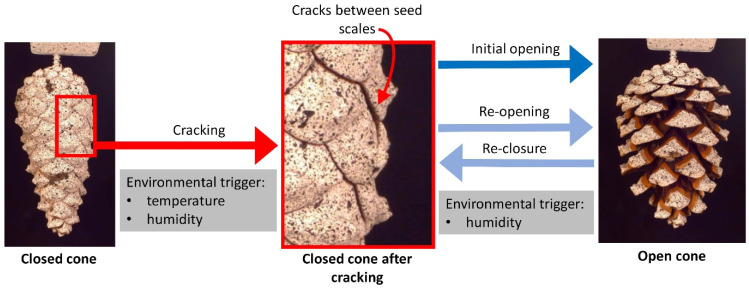
Movement processes in pine cones (here: *Pinus sylvestris*). The initially closed cone is sealed with resin, which is situated between the individual seed scales (left image). Cracking of the seal is initiated by high environmental temperature (because of the temperature-dependent viscosity of the resin changing from a solid state to a viscous liquid) and low humidity (the desiccated seed scales start to bend) (middle image; detail of red rectangle in left image). After cracking, the desiccated seed scales can fully bend, entailing the initial opening of the cone (right image). Once opened, the cone can undergo repetitive re-closure (under wet conditions) and re-opening events (during dry conditions). Note that the depicted cone is spray painted with a speckle pattern for 3D deformation analysis [18].

The hygroscopic opening and closing movements of pine cones attributable to the bending deformation of their seed scales have been studied intensively over the last few decades [1,2,3,4,5,6,7,8]. The seed scale movement is generally accepted to be based on a swelling (during uptake of water under wet environmental conditions) and shrinking (during evaporation of water during dry conditions) mismatch between various tissues, predominantly the sclereid and sclerenchyma layers. In addition to this functional bilayer actuation, current research has examined the hydraulic and mechanical properties of these layers [9,10] and has revealed that the isolated sclerenchyma fiber layer and the sclereid layer show hygroscopic movement. Furthermore, the 3D multiphase deformation of scales [11], the influence of delamination on scale movement in cyclically actuated pine cones [12], and the optimal timing of initial cone opening [13,14,15,16,17,18] have been investigated (Figure 1). Whereas the previous literature has described the arrangement of scales on the cone axis as a Fibonacci sequence, their interrelated movement sequences resulting from changes in environmental humidity [18,19,20] and the forces that they can produce have been little studied [9,18,21]. The relationship between the size of a motile plant structure, the time scale of the water displacement inside this structure, and the duration of movement was described by [22].

**Figure 2:**
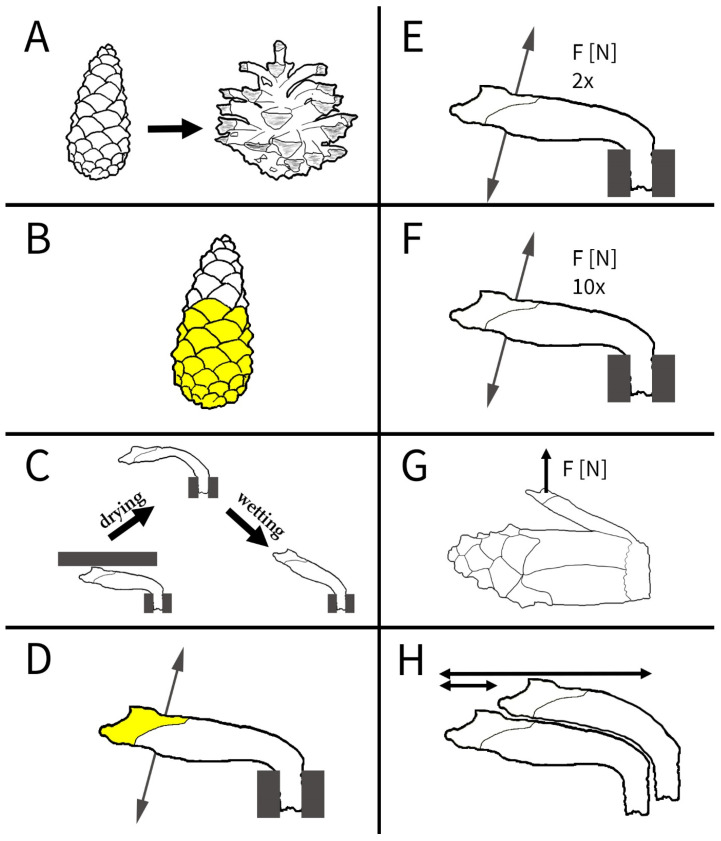
Schematic overview of the experiments conducted in this study to shed light on the orchestration of drying-induced pine cone opening and wetting-induced closing. (**A**) Analysis of the sequence of scale movement leading to cone opening. (**B**) Opening sequence of cones manipulated with Vaseline (yellow) on various regions of the outer scale surfaces, i.e., the apophyses. (**C**) Scales hindered in their movement during drying show altered morphologies (i.e., severe deformation) and regained their original shape during wetting, thereby showing shape-memory-like effects. (**D**) Movement of scales with different regions of the surfaces being manipulated with Vaseline. (**E**) Comparative force measurements in twice and (**F**) tenfold repeated movements. (**G**) Forces required to bend scales of a closed cone outwards. (**H**) Overlapping regions of scales.

To shed light on the desiccation-induced opening and wetting-induced closing processes in pine cones, we performed a series of interlinked experiments with cones and their scales from various pine species (Figure 2). First, we investigated the opening processes of cones. We manipulated them as a whole and as individual scales by selectively blocking the evaporation and uptake of water. Next, we measured the forces that individual scales can produce during drying in repetitive trials. We furthermore investigated the morphological overlap between scales in wet closed cones and measured the forces required to bend the scales outwards. As this is relevant in the ecological context of seed predation [23,24,25,26], our research was also aimed at a better understanding of the ecology of pines.

With the described experiments, we test the assumption that the abaxial scale surfaces are most relevant for water exchange during the hygroscopic movements, although the apophyses are in contact with the environment in both the open and closed states, and the scales are expected to overlap to a large extent. Furthermore, we proposed that the scales can withstand desiccation-induced deformation and recover their shape and biomechanical properties by subsequent wetting. Based on the predation observed on pine seeds, we also hypothesized that the pine cone scales are very tightly closed, thus thwarting predation attempts. The envisaged results concerning the actuation and mechanics of pine cones and their functional robustness and resilience might help to improve biomimetic actuators [27].

## 2. Methods

### 2.1. Kinematic Analyses of Cones

#### 2.1.1. General Procedures and Settings for Video Analyses of Whole Cones

We used already opened cones, i.e., collected after natural initial opening for our experiments. These cones were typically sized respective to the species. After being wetted in tap water, cones were dried for up to 6 days (until full opening) at 24 ± 1 °C and a relative humidity of 45 ± 3%. Up to three USB cameras (acA2040-90uc/acA2040–90um, Basler AG, Ahrensburg, Germany) with objective lenses (LM8HC 8 mm fish eye, LM35HC 35 mm, Kowa Company, Ltd., Nagoya, Japan) were used and controlled via the Basler pylon camera software suite to record these processes. Time lapses were recorded with 1 frame per 5 min. Two cameras recorded the cones from lateral views, and a third camera recorded the cones from top view.

#### 2.1.2. Experiment A: Unmanipulated Cone Opening

Cones of *Pinus jeffreyi* (4 cones), *P. nigra* (3 cones, both from trees in the Botanical Garden Freiburg, Germany), *P. sylvestris* (4 cones, from Essen, Germany), and *P. wallichiana* (2 cones, from Gelsenkirchen, Germany) were submerged in tap water overnight, leading to full closure. Screws for fixation were then drilled into the basal central axes of the cones. The opening of the cones was recorded with the above setup and settings (Figure 2A).

#### 2.1.3. Experiment B: Manipulation of Cone Surfaces with Vaseline

The simultaneous drying of eight *P. sylvestris* cones was recorded in the same preparations and under the identical environmental conditions and recording setup as described above. Initially, the opening processes of all cones were recorded in the unmanipulated condition. We then applied Vaseline to the re-wetted and therefore closed cones according to the scheme described in Table 1 to prevent water evaporation from certain areas of the cones. Again, the opening during drying was recorded as specified above (Figure 2B).

**Table 1:** Cone manipulation with Vaseline to analyze the relevance of various cone areas for evaporation and mechanistic functions. See also Figure 3.

Cone	Treatment
c1	Distal 2/3 region of cone covered with Vaseline
c2	Cone completely covered with Vaseline
c3	Basal 2/3 region covered with Vaseline
c4	Basal 1/3 region covered with Vaseline
c5	One half of the cone (longitudinal) covered with Vaseline
c6	Cone completely covered with Vaseline, except for four connected central scales
c7	Cone completely covered with Vaseline, except for a helical row of scales
c8	Cone completely covered with Vaseline, except for a central horizontal row of scales

### 2.2. Kinematic Analyses of Scales

#### 2.2.1. General Procedures and Settings for Video Analyses of Scales

For these analyses, we used *P. sylvestris* scales gently pulled out from the cone axes by hand from the central regions of typically sized, already naturally opened cones, taking care not to damage their surfaces, especially in the basal region, which is responsible for (most of the) hygroscopic motion [9]. Scales were then clamped at their bases by using curtain clips and filmed during wetting- and drying-induced bending with the acA2040-90uc USB camera and a 35 mm objective lens (LM35HC) as described above and via the pylon camera software suite. We recorded the movements of scales laterally and subsequently measured angular changes during wetting in tap water and subsequent drying at a temperature of 24 ± 1 °C and a relative humidity of 45 ± 3% via time-lapse, with 1 frame per minute during hydration and 1 frame per 5 min during dehydration. The video length was adjusted to the duration of movement in each experiment.

#### 2.2.2. Experiment C: Shape-Memory-like Effects in Scales

**Figure 4:**
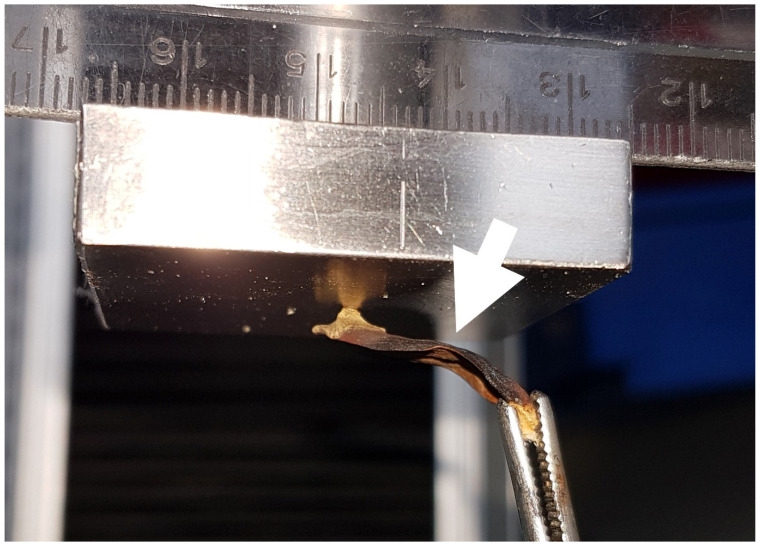
An initially wet scale pressing against a force transducer during drying. Note the kink after drying in the scale (white arrow; see shape-memory-like effects analysis). With this setup, we also measured the forces produced by the scale.

For this experiment, we hydrated scales of three cones overnight and then prevented their drying-induced motion by mechanical blocking. For this, we fixed the scales at their bases by using tweezers clamped in a vice and placed a force transducer (but the force was not recorded) in the “drying path” (Figure 4). After being dried, the thus prepared and slightly bent scales (n_bent_ = 12) were again placed under water, and the angular changes during wetting were measured (Figure 2C). A second set of scales (n_control_ = 12) remained unmanipulated (i.e., no mechanical blocking was applied) and was recorded for comparison.

#### 2.2.3. Experiment D: Manipulation of Scale Surfaces with Vaseline

For analyzing scale movements in relation to the water uptake and release through the scale surfaces, we covered various regions of the scales of three cones with Vaseline (Table 2) and recorded time lapses during the uptake of water and, subsequently, during drying (Figure 2D). Environmental conditions and setups were as described above. Subsequently, the angular changes and duration of the processes were compared.

### 2.3. Force Measurements

#### 2.3.1. Experiment E/F: Repeated Blocking Force Measurements

Wet *P. sylvestris* scales from three cones, incubated in tap water overnight, were tightly fixed by using tweezers and a heavy bench vice and were placed below a force sensor (static load cell, ±10 N; Instron GmbH, Darmstadt, Germany) (Figure 4). The scales were oriented directly beneath the force sensor so that, during drying for 6–8 h, the scales were pressed against it, and the respective forces could be measured. We conducted two experiments, a two-fold repetition on five scales (experiment 5) (Figure 2E) and a ten-fold repetition on two scales (experiment 6) (Figure 2F). All scales were weighed in their dry and wet states with a precision balance (Kern ABT 220-5DM, Balingen, Germany), and the relative maximum forces (normed by dry mass) were determined and compared among the different trials. For twice repeated blocking force measurements (experiment 5), we used five scales from three different cones, whereas, for the ten-fold repeated blocking force measurements (experiment 6), we used two scales from two different cones.

The maximal forces achieved per run were determined and compared between the repetitions. We compared the forces produced between scales statistically and plotted the observed maximal forces to disclose potential patterns. We compared absolute and relative (normed by weight) forces.

#### 2.3.2. Experiment G: Forces in the Closed Cone

We measured the forces necessary to pull single *P. sylvestris* scales in the wet closed cone condition away from the cone axes, simulating the force of a potential seed predator bending the scales. Therefore, we removed all basal scales of a cone. Thus, the bases of up to four scales in the center of each cone lay open (Figure 2G). The scale axes were tightly clamped in a vice. We then drilled small holes through the middles of the apophyses in each scale (n = 11, from 4 cones) by using a 1 mm drill and attached thin nylon threads at a 90° angle to the apophysis between the scales and the force sensor (static load cell, ±10 N). We measured the force increase occurring during pulling with a pulling speed of 1 mm/min until 2 N was reached to keep the deformation elastic. A plastic deformation would prevent the subsequent step. Subsequently, the scales distal of the investigated scales were removed, and the measurements were repeated. Because of the damage to some of the investigated scales caused by the mechanical removal of distal scales after the first experimental run (handling artifacts, cracks), some scales had to be excluded from the analysis with removed distal scales (n = 8, from 3 cones).

### 2.4. Experiment H: Morphological Overlap Measurements

We analyzed the morphological overlap of scales in the central region, i.e., to what degree scales were covered by the neighboring scales, by carefully sawing 9 wet closed cones into two halves with a Japanese pull saw. We then photographed the cone halves on graph paper and measured the lengths of the apophyses and the total scale lengths (Figure 2H). Total lengths were determined as polylines beginning at the insertion at the cone axis and ending at the scale tips. The lengths of the apophyses were measured as longitudinal apophysis lengths parallel to the measurement of the scale lengths (Figure 2H).

### 2.5. Statistics

All statistical analyses were carried out using R 4.1.2 [28]. Normally distributed data were analyzed using *t*-tests. Non-parametric data sets were analyzed using Wilcoxon tests for pair-wise comparisons.

## 3. Results

### 3.1. Experiment A: Repetitive Cone Opening and Scale Movement Orchestration

**Figure 5:**
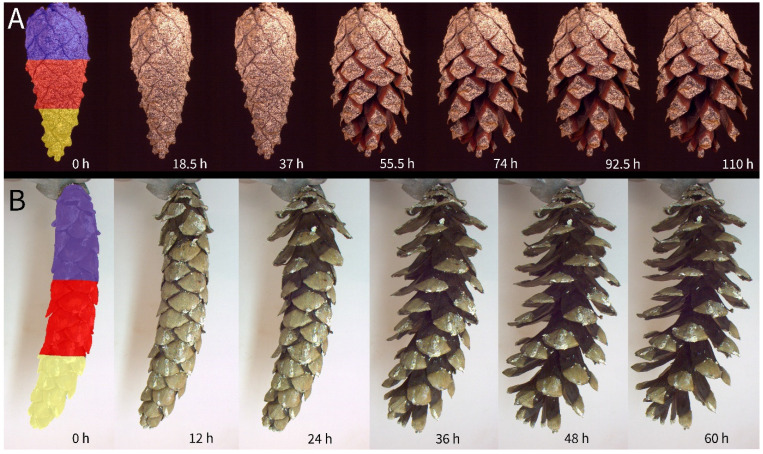
Opening of characteristic cones of *P. sylvestris* (**A**) and *P. wallichiana* (**B**). Regions defined as the basal (purple), central (red), and apical parts (yellow) of a cone are color-coded. Note that the depicted cone of *P. sylvestris* has been spray-painted with a speckle pattern for 3D deformation analysis ([18]).

The repetitive desiccation-driven cone opening showed a recurring pattern in all investigated species. First, the drying of the cone surfaces was accompanied by a slight shrinking (see, for example, Supplementary Videos S2 and S3), followed by the opening of the cone. All cones started to open at the most basally arranged scales, followed consecutively by the scales situated more distally (Supplementary Videos S1–S4, Figure 5). In comparatively large cones, like those of *P. wallichiana*, the basal part could therefore be nearly fully opened (i.e., the scales have achieved their maximum bending angles), while the apical part remained closed. The last scales of the apical region of the cones opened nearly simultaneously in all species. A strict opening in a Fibonacci sequence was never observed. The opening was often delayed by an initial drying phase, as the cones had been soaked in water, but once dry, cone opening took place relatively quickly.

### 3.2. Experiment B: Manipulated Repetitive Cone Opening

**Figure 6:**
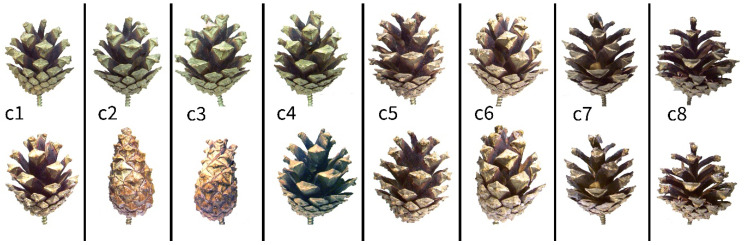
Effects of various manipulative experiments with Vaseline on the drying-induced opening of *P. sylvestris* cones. **Top** row: Eight dry and open cones. **Bottom** row: The same cones as in the top row, but after having been fully wetted until full closure, manipulated with Vaseline in various ways (c1–c8), and then left to desiccate in a dry environment for over 100 h. Bottom row, from left to right: apical 2/3 of the cone covered with Vaseline (c1), cone completely covered (c2), basal 2/3 of the cone covered (c3), basal 1/3 of the cone covered (c4), left cone half covered (c5), cone completely covered other than four central scales (c6), cone completely covered other than a diagonal row of scales (c7), cone completely covered other than a horizontal row (see also Figure 3 and Table 1).

The various manipulative treatments of wet cones with Vaseline had different effects on their motion behavior in a dry environment (Figure 6). Completely covering the cone surface with Vaseline delayed the cone opening drastically. The unmanipulated cones opened simultaneously within 2 days. Covering one of these cones completely with Vaseline delayed opening by 4 days to a total duration of 6 days. Covering the apical and central parts of another cone’s surface, however, slowed down the opening of these parts by about 16 h, while the basal part opened normally (Supplementary Video S5).

Covering the basal and central part of a cone’s surface slowed down cone opening even more. Only the most apical scales appeared to bend outwards slightly during the first two days. These cones did open, but beginning at the cone’s tip, i.e., the opposite of the typical opening sequence. Opening in these cones was completed after about 110 h. Covering the basal part of the cone surface initially also had a retarding effect, but after 48 h, cone opening was comparable to that of the unmanipulated cones. Blocking the cone in this way did not lead to distinct alterations of the opening sequence (Supplementary Video S6).

### 3.3. Manipulated Repetitive Scale Movement

#### 3.3.1. Experiment C: Shape-Memory-like Effects of Scales

**Figure 7:**
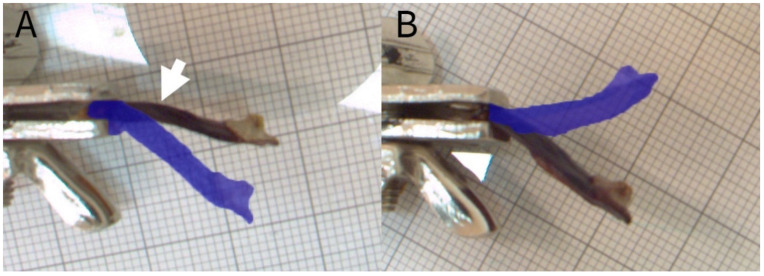
Shape memory of deformed scales. The true color images give the start configuration, whereas the blue-shaded overlay gives the final position after each process. (**A**) Wetting-induced bending of a kinked (white arrow) scale. (**B**) Drying-induced bending of the same scale after wetting.

**Figure 8:**
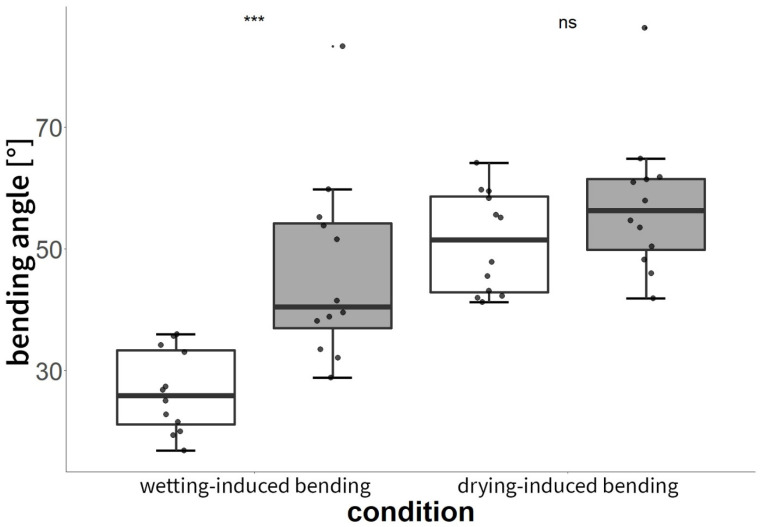
Boxplots showing bending angles of untreated (white, n = 12) and manipulated (blocked, deformed) scales (gray, n = 12). Manipulated scales were mechanically blocked in their motions during their foregoing desiccation-induced motions and exhibited significantly different bending reactions during subsequent wetting. In the subsequent actuation cycle by drying, bending angles no longer showed significant differences. *** = *p* < 0.001, ns = *p* > 0.05.

*P. sylvestris* scales that were blocked by an obstacle during their drying-induced motions showed conspicuously kinked and deformed shapes (Figure 4 and Figure 7A). Whereas untreated scales of *Pinus sylvestris* typically performed a bending deformation of 46 ± 15°, manipulated (deformed) scales showed significantly reduced angles of 26 ± 6° (*t*-test, t = −4.0983, df = 15.14, *p* < 0.001, n = 12, Figure 7 and Figure 8, Supplementary Video S7) upon wetting. The subsequent drying-induced bending reactions with angular changes of 54 ± 10° were not significantly altered (*t*-test, t = −1.4872, df = 20.039, *p* > 0.05, n = 12) and the kinked and deformed shapes were no longer visible.

#### 3.3.2. Experiment D: Manipulation of Isolated Scales with Vaseline

*P. sylvestris* scales showed different bending responses depending on the type of surface manipulation with Vaseline (Table 3). All unmanipulated scales moved simultaneously when immersed in water and conducted their wetting-induced bending deformation in 118 ± 25 min (Supplementary Videos S8 and S9). The drying of unmanipulated scales and their respective bending deformation took 903 ± 259 min. However, covering the scale surfaces (partly) with Vaseline slowed down all movements but without alterations in the final angles reached by the scales. Whereas covering the adaxial scale sides increased the wetting-induced bending duration by 221 min, the drying-induced bending took 256 min longer. Covering only the apophyses with Vaseline had similar effects as covering the entire adaxial sides. The wetting-induced bending duration was increased by 245 min, and the drying-induced bending by 491 min. Covering the abaxial side, but not the apophysis, with Vaseline led to an increase in the wetting- and drying-induced bending by 446 min and 1356 min, respectively. Repeating this treatment by covering the whole abaxial side (including the apophysis) with Vaseline increased the wetting-induced bending duration even more by 664 min. The bending during drying took 725 min longer, which is less than the drying-induced bending duration with just the apophysis being covered. Covering the whole scale surfaces with Vaseline, but not the apophyses, increased the wetting-induced bending duration by 1249 min, whereas drying-induced bending took 2413 min longer. Smearing the whole scale surface with Vaseline retarded the wetting-induced movement by 2037 min, whereas the drying-induced movement was retarded by 5850 min, which represented the largest observed increase in movement duration.

### 3.4. Blocking Force Measurements

#### 3.4.1. Experiment E: Twice-Repeated Blocking Force Measurements

**Figure 9:**
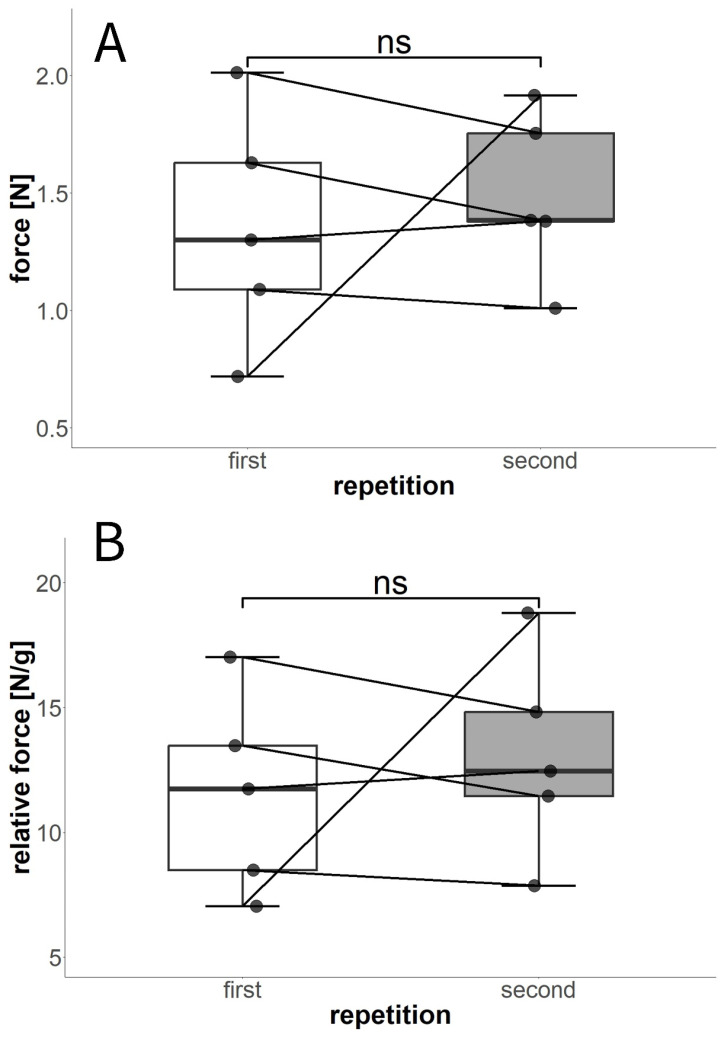
Twice-repeated blocking force measurements of drying-induced scale deformation. (**A**) Comparison of absolute forces generated. (**B**) Comparison of forces relative to the weight of the scales. The lines indicate the pairs of values from the same scales (n = 5). ns = *p* > 0.05.

*P. sylvestris* scales in the twice-repeated measurement weighed between 0.10197 g and 0.12833 g. We found no significant alterations between the initial forces measured and during the follow-up repetitions (Figure 9). Some of the scales showed lower forces during the repetition, whereas some showed higher forces. However, these results are independent of the absolute or relative force values. One scale showed the lowest force measured in the first experiment and the highest of all forces in the second experiment. Overall, the forces were slightly higher during the repetitions, but these differences were not significant (Wilcoxon test, W = 10, *p* = 0.69).

#### 3.4.2. Experiment F: Tenfold-Repeated Blocking Force Measurements

**Figure 10:**
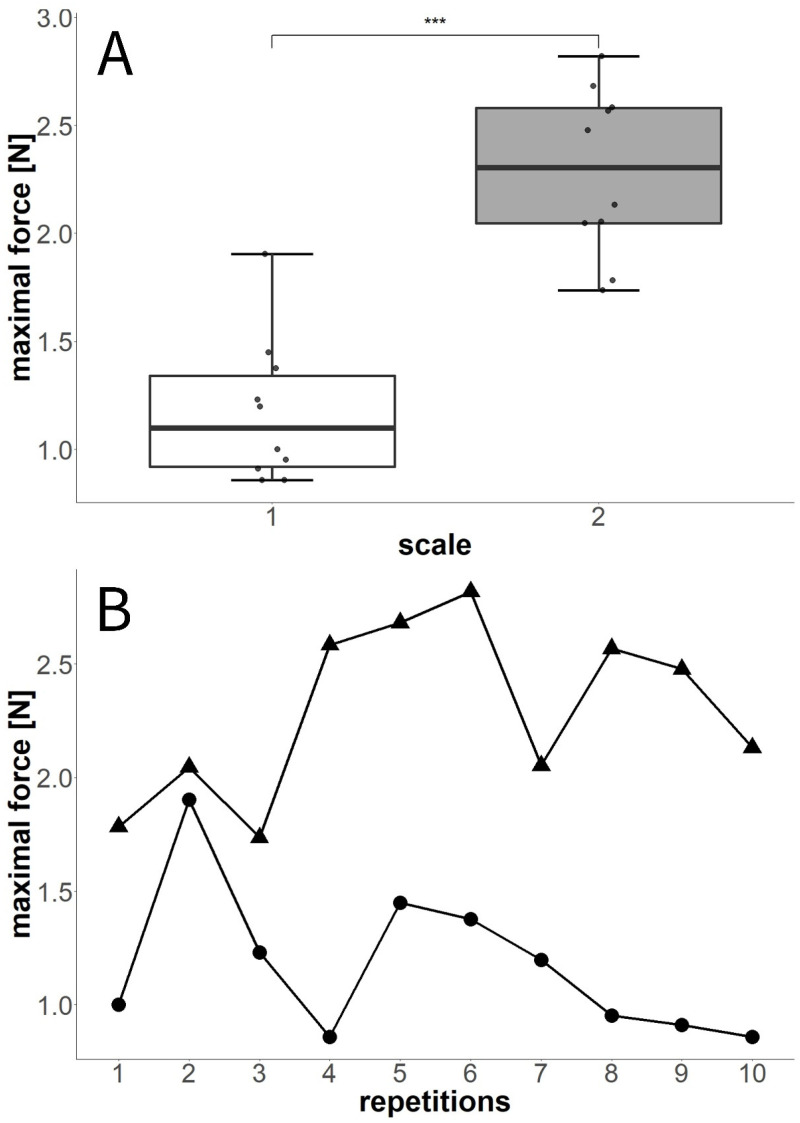
Maximum blocking forces measured in two scales during tenfold successive desiccation processes. (**A**) Statistical comparison between scale 1 and scale 2. The values measured during the repetitions were highly significantly different between scales. (**B**) Maximum forces measured for each of the scales (circles = scale 1, triangles = scale 2) during the 10 repetitions. *** = *p* < 0.001.

In the tenfold-repeated blocking force measurement, *P. sylvestris* scale 1 weighed 0.20709 g and scale 2 weighed 0.20530 g. The ten-times-repeated force measurements (Figure 10) revealed significant differences between the two scales tested (Wilcoxon test, W = 2, *p* < 0.001). The average maximum values measured for each scale were 1.11 ± 0.42 N (scale 1) and 2.29 ± 0.39 N (scale 2). In both scales, forces higher and lower than the initially observed value were recorded during the experiment. Although, in one scale, the maximum force was reached as early as the second repetition, the second scale attained this force during the sixth movement. Overall, no striking pattern concerning an increase or decrease in maximum force over time was recognizable, although forces in scale 1 were successively reduced after the fifth repetition (Figure 10B).

### 3.5. Experiment G: Forces in the Closed State

**Figure 11:**
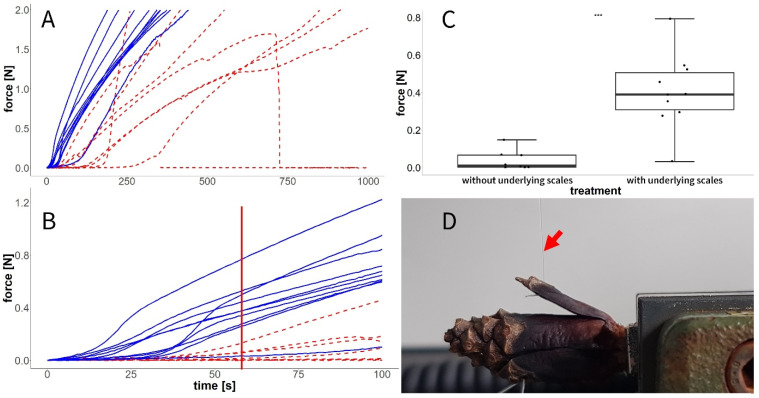
Forces necessary to pull open wet cone scales with (blue, solid line) or without (i.e., removed) underlying scales (red, dashed line). (**A**) Full experiment over 1000 s. Measurements were stopped at 2 N. (**B**) Detail from the data presented in (**A**) showing the first 100 s of the experiment. The line denotes the 1 min mark described in (**C**). (**C**) Statistical comparison of the forces occurring 1 min after the start of the experiment (red line in (**B**)). (**D**) Setup for measuring the closing force of the scales by using a nylon thread (red arrow) connected to a force transducer. *** = *p* < 0.001.

We found that forces during our pulling tests were constantly higher in those *P. sylvestris* scales in which the underlying scales of the cone were not removed (Figure 11A). This was especially noticeable in the first 100 s of the experiment (Figure 11B).

A comparison of the forces after 60 s revealed that the treatment with removed underlying scales significantly decreased forces (Wilcoxon test, W = 3, *p* ≤ 0.001, Figure 11C) when the scales were pulled outwards (Figure 11D).

### 3.6. Experiment H: Overlap Measurements

We found that scales situated centrally on the closed cone overlapped by about 71% ± 4.5% (MIN: 62%, MAX: 80%, n = 32 (9 cones)), i.e., ~30% of the length of the scales was represented by the apophysis, which had direct contact with the environment.

## 4. Discussion

We observed that the basal scales moved first during desiccation, thereby exposing the abaxial surfaces of the more apical scales to the environment. This is in accordance with prior observations [4,29,30] and with studies on the water uptake properties of the ab- and adaxial scale surfaces [5,9]. The manipulation experiments with Vaseline established that the abaxial surface of the scales in the *P. sylvestris* cones was responsible for most of the water release or uptake, as the covering of these surfaces extended the respective movement times drastically. Therefore, the opening of the whole cone is fundamentally based on the successful actuation of the basal scales and, subsequently, of the next scale rows and can be completely inhibited (or, at least, drastically prolonged) by blocking the basal scales.

Often, the most apical scales appear to open simultaneously. This is probably influenced by the diffusion of moisture towards the cone axis and into the dry basal part causing the observed simultaneous opening of scales close to the apex [31]. Furthermore, the most apical scales often seem to close not as tightly as the more basal ones, potentially exposing parts of the abaxial scale surfaces (Figure 1). Since the repetitive opening of cones has previously been shown not to slow down the opening movement under constant environmental conditions [11], the measured retardation is caused by the manipulation with Vaseline and is not attributable to the repetition of opening and closing.

In our investigations regarding which regions of the scale surface or which parts of the scale are the most important for bending actuation, we noticed that the covering of the apophyses with Vaseline had no distinct effect on the duration of scale movements. These findings suggest that the apophyses play only a minor role in water exchange with the environment. Covering the abaxial surfaces without the apophyses extended the movement durations whereas covering the whole abaxial surfaces (including the apophyses) extended the wetting-induced movement durations even more. Smearing the whole abaxial surface with Vaseline led to shorter desiccation-induced movement durations than smearing the whole abaxial surface without the apophyses. At present, we only can hypothesize that previously unknown direction-dependent diffusion and/or evaporation processes are responsible.

When the adaxial surfaces were covered with Vaseline, the changes in wetting- and drying-induced bending duration were negligible. Our data suggest that the abaxial surface is especially important for the uptake and evaporation of water, whereas the water uptake and evaporation on the adaxial surface, which was covered in this experiment, have only marginal effects. The results of this manipulation experiment support the idea that the apophysis has little influence on water exchange between the scale and its environment. Leaving only the apophysis uncovered and covering the whole remaining scale with Vaseline approximately doubled the wetting- and desiccation-induced movement durations. These results further support the marginal relevance of the apophyses for water exchange with the environment. This is probably attributable to the increased amount of alpha- and beta-pinene and of oleoresin or other components in this structure [30,32].

As the apophyses are by far the dominating structural element on the closed cone surface, they probably inhibit cone opening by their barrier function. In the very basal scales, the apophyses represent a large proportion per scale (Figure 11A). Therefore, the opening is decelerated until the first abaxial surfaces of the scales are exposed to the environment. Scales of the central cone overlap by about 70%, i.e., 30% of the scale is represented by apophyses in these central scales (Figure 11B). Cone opening is only initialized once the abaxial sides of these scales are exposed (Figure 5).

**Figure 12:**
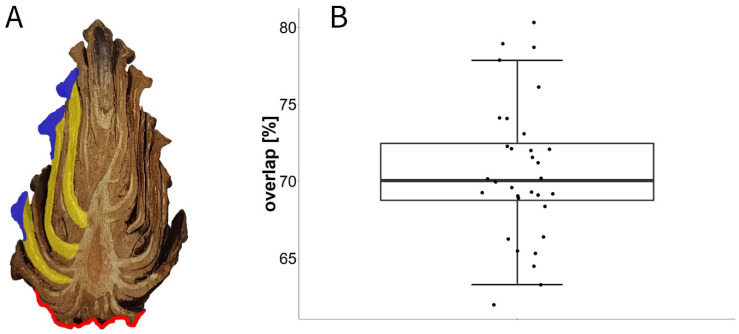
Overlap of scales in the wet closed *P. sylvestris* cone. (**A**) We assessed the overlap of scales by measuring the distances from the insertions of the scales to the tips of the scales as polylines. The apophysis is marked blue, while the rest of each scale is marked yellow. The red line shows the area, in which the surfaces of the small basal-most scales consist nearly completely of their apophyses. (**B**) Analysis of overlap in percent of scale length in 32 scales from 9 cones (3–5 per cone) to determine the average overlap between scales.

The importance of the abaxial surfaces of the scales and of their basal regions for the initiation of cone opening also becomes obvious in the Vaseline manipulation experiments on whole cones. Covering the basal and central parts of cones with Vaseline (thereby inhibiting the weak water exchange with the environment via the apophyses) had similar effects to those of covering the whole cone. Presumably, the initial evaporation of water from the cones takes place in this region. Covering only the basal part of the cone with Vaseline also led to a slightly prolonged desiccation-induced movement but with a retardation of only a few hours. Furthermore, covering the central and apical parts of the cones decelerated the cone opening only moderately by 16 h. Here, the basal part opened in a normal fashion, whereas the opening sequence of the apical region was slowed down. This supports the idea that the basal and central scales or their apophyses are most relevant for initial evaporation before cone opening, whereas exactly these apophyses show the slowest evaporation of all scale regions. Investigation of sections of a closed cone (Figure 12A) reveals that the insertions of even the central scales are positioned in the basal half of the cone and are therefore affected by the opening of the very basal scales.

Regarding the closed configuration with overlapping scales, previous studies had not clarified whether the wetted scales are “mechanically inert” or whether they exert force on the underlying (more apical) scales and thwart, for example, seed predators that try to bend the scales open [23,33]. In our experiments, we found that, in closed cones with removed distal scales, the force necessary to pull scales away from the cone axis was smaller than in cones in which these scales were in contact with scales below. This establishes that the scales of a wet cone press against the underlying scales in the next row. From an ecological viewpoint, the cones shut tight and keep this configuration until seed dispersal conditions are suitable [6,14,18,34,35,36]. The higher forces required to bend the scales of an intact cone presumably reduce the likelihood that seeds can be extracted by animals, e.g., crossbills or squirrels [23,33,37]. This protective function is presumably increased even more by the resin that “glues” neighboring scales together in immature cones before their first opening and furthermore acts as a temperature-sensitive chemical-mechanical latch for this initial cone opening [18]. This temperature-sensitive, chemical–mechanical latch is further steered by the mechanisms found and helps pines to adjust their seed release to environmental conditions, which should be considered in future investigations, e.g., on serotinous pine species [38,39,40,41].

Scale movement is highly reproducible in terms of general kinematics. We have observed differences regarding the respective blocking forces produced during repetitions but without any specific pattern. Within the two-fold-repeated experiment, no tendency towards a decrease or increase in forces was observed (see also [12] for *Pinus jeffreyi* scales). Moreover, in the maximally 10-times conducted repetitions, we also found no clear pattern, neither a reduction nor an increase in forces.

This resilient and robust opening and closing mechanism is probably a by-product of the morphology necessary to fulfill the task of seed production, protection, and release (see Supplementary Videos S10 and S11) under favorable environmental conditions [14,18,21,34,35]. Future studies should be carried out to determine to what extent the repeated opening and closing of the cone is of ecological or adaptive significance. Extremely high functional robustness “beyond their biological purpose” is otherwise known, for example, from fossil cones [42] and the motile petals of the bird of paradise plant (*Strelitzia reginae*) [43].

Our experiments have shown that scales that are deformed by the blocking of their drying-induced movement path can be reshaped within one wetting–drying cycle. We assume that this “shape-memory-like behavior” of scales is more of a consequence of the morphological and structural composition than of an ecologically relevant function. Probably, a molecular recovery mechanism in the cell matrix exists, as otherwise shown by [44] during the tensile deformation of various wood tissue types. Our observations confirm the existence not only of the high functional robustness in pine cones and their scales, which can move even after millions of years [42] but also of their functional resilience [45]. Interestingly, cone scales may show high morphological plasticity [46], and it would be interesting to evaluate how this interferes with the here observed robustness/resilience.

As an outlook, these functions can serve as models and concept generators for the development of sustainable and resilient biomimetic components for engineering and architectural applications [11,43,47,48,49,50].

## Figures and Tables

**Figure 3:**
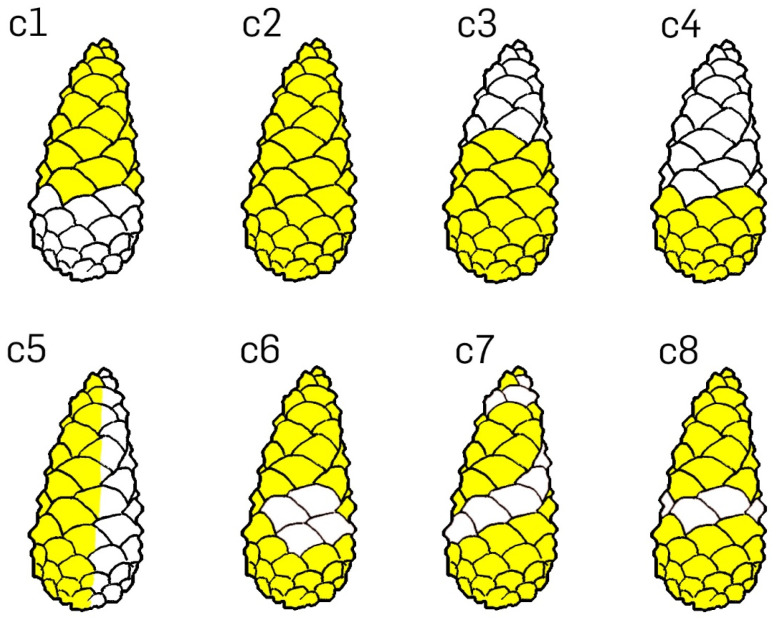
Cone manipulation with Vaseline. Eight schematic closed cones (c1–c8) are shown with certain scales or regions of scales having been covered with Vaseline (yellow) and with untreated scales or regions of scales (white). See description in Table 1.

**Table 2:** Scale manipulation with Vaseline.

Scale Set	Treatment
s1	Whole abaxial surface covered
s2	Abaxial surface covered except for the apophysis
s3	Whole adaxial surface covered
s4	Scale completely covered
s5	Scale completely covered except for the apophysis
s6	Only apophysis covered

**Table 3:** Experimental surface manipulations in *P. sylvestris* scales with Vaseline and wetting- and drying-induced scale bending durations. Respective standard deviations and differences from unmanipulated scales (Δ) are also given.

Treatment with Vaseline	n	Unmanipulated		Manipulated		Difference Δ between Manipulated and Unmanipulated Scales	
		Wetting-Induced Bending (min)	Drying-Induced Bending (min)	Wetting-Induced Bending (min)	Drying-Induced Bending (min)	Wetting-Induced Bending [min]	Drying-Induced Bending [min]
No manipulation	12	118 ± 25	903 ± 259	-	-	-	-
Adaxial scale surface covered	4	110 ± 22	981 ± 321	331 ± 66	1238 ± 43	221	256
Surface of apophysis covered	3	130 ± 28	758 ± 29	375 ± 25	1250 ± 43	245	492
Abaxial scale surface covered, but not apophysis	4	141 ± 13	650 ± 29	588 ± 75	2006 ± 783	446	1356
Abaxial scale surface covered	4	86 ± 18	1075 ± 140	750 ± 65	1800 ± 767	664	725
Scale surface covered, but not apophysis	4	126 ± 6	738 ± 48	1375 ± 261	3150 ± 0	1249	2413
Scale surface entirely covered	3	113 ± 24	1275 ± 152	2150 ± 520	7125 ± 130	2037	5850

## Data Availability

All relevant data are included within the paper and its Supplementary Material files.

## Supplementary Materials

The following supporting information can be downloaded at: https://zenodo.org/records/11952704 (accessed on 17 June 2024), Video S1: Opening of *Pinus sylvestris* cone in lateral view. Video S2: Opening of *Pinus nigra* cone in top and lateral view. Video S3: Opening of *Pinus jeffreyi* cone in top and lateral view. Video S4: Opening of *Pinus wallichiana* cone in top and lateral view. Video S5: Manipulation of cone opening of *Pinus sylvestris* with Vaseline. First set of cones. Video S6: Manipulation of cone opening of *Pinus sylvestris* with Vaseline. Second set of cones. Video S7: Shape-memory-like effects in kinked and unkinked scales of *Pinus sylvestris* in water. Video S8: Effects of covering different scale regions with Vaseline to block water uptake of *Pinus sylvestris* in water. First set of scales. Video S9: Effects of covering different scale regions with Vaseline to block water uptake of *Pinus sylvestris* in water. Second set of scales. Video S10: Seed release from a cone of *Pinus sylvestris* after drying-induced opening. First seed release event. Video S11: Seed release from a cone of *Pinus sylvestris* after drying-induced opening. Second seed release event.
